# Risk factors for acquisition of meningococcal carriage in the African meningitis belt

**DOI:** 10.1111/tmi.13203

**Published:** 2019-02-06

**Authors:** Laura V. Cooper, Anna Robson, Caroline L. Trotter, Abraham Aseffa, Jean‐Marc Collard, Doumagoum Moto Daugla, Aldiouma Diallo, Abraham Hodgson, Jean‐François Jusot, Babatunji Omotara, Samba Sow, Musa Hassan‐King, Olivier Manigart, Maria Nascimento, Arouna Woukeu, Daniel Chandramohan, Ray Borrow, Martin C. J. Maiden, Brian Greenwood, James M. Stuart, the MenAfriCar Consortium, Oumer Ali, Ahmed Bedru, Tsehaynesh Lema, Tesfaye Moti, Yenenesh Tekletsion, Alemayehu Worku, Haimanot Guebre Xabher, Lawrence Yamuah, Rahamatou Moustapha Boukary, Ibrahim Dan Dano, Ibrahim Habiboulaye, Bassira Issaka, Sani Ousmane, Issoufa Rabe, Jean Pierre Gami, Kadidja Gamougam, Lodoum Mbainadji, Nathan Naibei, Maxime Narbé, Jacques Toralta, Abdoulaye Berthe, Kanny Diallo, Mahamadou Keita, Uma Onwuchekwa, Samba O. Sow, Boubou Tamboura, Awa Traore, Alou Toure, Tom Clark, Leonard Mayer, Mary Amodu, Omeiza Beida, Galadima Gadzama, Zailani Sambo, Shuaibu Yahya, Brian M. Greenwood, Nicole E. Basta, Xilian Bai, Helen Findlow, Serge Alavo, Hubert Bassene, Marietou Dieng, Souleymane Doucouré, Jules François Gomis, Assane Ndiaye, Cheikh Sokhna, Jean François Trape, Bugri Akalifa, Abudulai Forgor, Isaac Osei, Stephen L. Quaye, John Williams, Peter Wontuo, Thomas Irving, Julia Bennett, Dorothea Hill, Odile Harrison, Martin C.J. Maiden, Lisa Rebbetts, Eleanor Watkins

**Affiliations:** ^1^ University of Cambridge Cambridge UK; ^2^ Armauer Hansen Research Institute Addis Ababa Ethiopia; ^3^ Centre de Recherche Médicale et Sanitaire Niamey Niger; ^4^ Bactériologie expérimentale Institut Pasteur de Madagascar Antananarivo Madagascar; ^5^ Centre de Support en Santé Internationale N'Djamena Chad; ^6^ Institut de Recherche pour le Développement Dakar Senegal; ^7^ Navrongo Health Research Centre Navrongo Ghana; ^8^ Centre de Recherche Médicale et Sanitaire Niamey Niger; ^9^ Department of Community Medicine University of Maiduguri Maiduguri Nigeria; ^10^ Centre pour les Vaccins en Développement Bamako Mali; ^11^ Faculty of Infectious and Tropical Diseases London School of Hygiene & Tropical Medicine London UK; ^12^ Public Health England Vaccine Evaluation Unit Manchester UK; ^13^ University of Oxford Oxford UK

**Keywords:** acquisition, risk factors, *Neisseria meningitidis*, Africa, acquisition, facteurs de risque, *Neisseria meningitidis*, Afrique

## Abstract

**Objective:**

To investigate potential risk factors for acquisition in seven countries of the meningitis belt.

**Methods:**

Households were followed up every 2 weeks for 2 months, then monthly for a further 4 months. Pharyngeal swabs were collected from all available household members at each visit and questionnaires completed. Risks of acquisition over the whole study period and for each visit were analysed by a series of logistic regressions.

**Results:**

Over the course of the study, acquisition was higher in: (i) 5‐to 14‐year olds, as compared with those 30 years or older (OR 3.6, 95% CI 1.4–9.9); (ii) smokers (OR 3.6, 95% CI 0.98–13); and (iii) those exposed to wood smoke at home (OR 2.6 95% CI 1.3–5.6). The risk of acquisition from one visit to the next was higher in those reporting a sore throat during the dry season (OR 3.7, 95% CI 2.0–6.7) and lower in those reporting antibiotic use (OR 0.17, 95% CI 0.03–0.56).

**Conclusions:**

Acquisition of meningococcal carriage peaked in school age children. Recent symptoms of sore throat during the dry season, but not during the rainy season, were associated with a higher risk of acquisition. Upper respiratory tract infections may be an important driver of epidemics in the meningitis belt.

## Abstract (fr)

**Objectif:**

Investiguer les facteurs de risque potentiels d'acquisition dans sept pays de la ceinture de la méningite.

**Méthodes:**

Des ménages ont été suivis toutes les deux semaines pendant deux mois, puis tous les mois pendant quatre mois. Des prélèvements pharyngés sur écouvillons ont été collectés auprès de tous les membres disponibles du ménage à chaque visite et des questionnaires ont été remplis. Les risques d'acquisition sur l'ensemble de la période d’étude et pour chaque visite ont été analysés par une série de régressions logistiques.

**Résultats:**

Au cours de l’étude, l'acquisition a été plus élevée chez: (i) les 5‐14 ans, par rapport à ceux âgés de 30 ans ou plus (OR = 3,6; IC95%: 1,4‐9,9); (ii) les fumeurs (OR = 3,6; IC95%: 0,98‐13); et (iii) les personnes exposées à la fumée de bois à la maison (OR = 2,6; IC95%: 1,3‐5,6). Le risque d'acquisition d'une visite à l'autre était plus élevé chez les personnes signalant un mal de gorge pendant la saison sèche (OR = 3,7; IC95%: 2,0‐6,7) et plus faible chez celles signalant une utilisation d'antibiotique (OR = 0,17; IC95%: 0,03‐0,56).

**Conclusions:**

L'acquisition du portage du méningocoque a culminé chez les enfants d’âge scolaire. Les symptômes récents de maux de gorge pendant la saison sèche, mais pas pendant la saison des pluies, étaient associés à un risque d'acquisition plus élevé. Les infections des voies respiratoires supérieures pourraient être un facteur important d’épidémies dans la ceinture de la méningite.

## Introduction

Epidemics of meningococcal meningitis occur periodically in the African Meningitis Belt, an area of sub‐Saharan Africa stretching from Senegal in the west to Ethiopia in the east 1. These epidemics are highly seasonal, with the majority of cases occurring during the dry season, predominantly in the first 5 months of the year 2. Given that asymptomatic pharyngeal carriage of meningococci is relatively frequent (ranging from 3% to 30% of the population) 3 and because meningococcal acquisition only occasionally leads to invasive disease, one explanation for this striking seasonality is an increased risk of invasive disease in the dry season, due to mucosal damage from environmental factors such as low absolute humidity and dust 1, 4, 5. Another hypothesis suggested by mathematical modelling is that higher rates of meningococcal transmission during the dry season, combined with population immunity, may be sufficient to explain epidemic patterns 6. Although a review of carriage in the meningitis belt published in 2007 found no evidence to support a seasonal effect on carriage 3, more recent studies have found a higher prevalence of carriage in the dry season 7, 8.

Studies of carriage prevalence and acquisition will, therefore, lead to a better understanding of the epidemiology of meningococcal meningitis in the African meningitis belt. The African Meningococcal Carriage Consortium (MenAfriCar) undertook 20 cross‐sectional carriage surveys in seven African meningitis belt countries from July 2010 to July 2012, involving the collection of over 48 000 pharyngeal swabs. These studies found a higher frequency of carriage in children aged 5 to 14 years, in the dry season and in rural populations 7. During these surveys, households with at least one pharyngeal carrier of *N. meningitidis* were recruited for longitudinal studies 9.

Previous longitudinal studies in the meningitis belt have been undertaken mainly at the population level 10, 11, 12 and few have investigated the transmission and acquisition of carriage at an individual level 13, 14. The aim of this MenAfriCar study was to investigate a comprehensive set of potential risk factors for the acquisition of carriage of *N. meningitidis* across the African meningitis belt.

## Methods

### Household surveys

Households included in this study were recruited during the course of cross‐sectional surveys conducted in seven countries in the African meningitis belt (Chad, Ethiopia, Ghana, Mali, Niger, Nigeria and Senegal) in 2010, 2011 and 2012. Details of the survey methods employed have been published previously 7. Longitudinal surveys were triggered by the identification of a putative carrier during a cross‐sectional survey (Visit 0). This initial identification of carriers relied on conventional microbiology and was later confirmed via molecular methods at the University of Oxford. In some cases, molecular methods did not confirm the presence of meningococci, so 51 of 184 households recruited to the study did not have an index carrier.

Within 4 weeks of the identification of a carrier, all members of the putative carrier's household were invited to take part in further studies (Visit 1). The head of the household was asked about characteristics of the household, including numbers of rooms and bedrooms, sleeping arrangements, location of kitchen and cooking fuel, house construction, drinking water, sanitation and household assets such as vehicle ownership, livestock and electrical goods.

A pharyngeal swab sample was obtained from all members of a household who gave consent and a questionnaire completed which included questions on: smoking; social activities; symptoms of recent respiratory tract infection; socio‐economic status and educational level; school attendance; travel history; recent medication including antibiotics; meningitis vaccination; and ethnic group. Carrier households were followed up 2‐weekly for 2 months (Visits 2–5) and monthly for a further four months (Visits 5–9). At each follow‐up visit, each household member was asked for a pharyngeal swab sample and to answer a short follow‐up questionnaire on factors that might have changed since the previous visit, such as symptoms of a respiratory tract infection.

### Laboratory methods

Pharyngeal swab samples, taken from the posterior pharynx and tonsillar fossa via the mouth, were plated directly onto Modified Thayer Martin agar plates in the field, taken to the laboratory within 6 h of collection and processed as previously described 9. A sample of boiled suspensions of Gram‐negative oxidase positive bacteria was sent to the University of Oxford for molecular analysis. Amplification and sequencing of the rplF gene was used to confirm the presence of, and to differentiate between, *Neisseria* species. Confirmed *N. meningitidis* were further characterised by genogroup (including capsule‐null) and porA genosubtype.

### Data management

Data were managed using the Teleform system version 10.4.1 (Autonomy, Cambridge, UK) with a separate database module linking the main study database with genetic laboratory results from the Oxford PubMLST.org/neisseria database (https://pubmlst.org/neisseria). Data from the longitudinal questionnaires were merged using a common person ID or census number, person matching was checked, any duplicate entries were removed and aberrant values excluded.

### Statistical analysis

The genogroup‐specific acquisition rates and 95% confidence intervals were calculated as Poisson rates, counting the number of acquisitions occurring in non‐index carriers and the time at risk as the days between the first carriage‐negative swab and the first positive swab. A series of fixed‐effects logistic regressions were used to identify significant risk factors for acquisition. In the first round of regressions, individual risk factors were included in a multi‐variable logistic regression with the *a priori* variables sex, age group and country. In the second round, risk factors with *P* < 0.1 in round 1 were added to a single model with *a priori* variables. In the third round, risk factors with *P* < 0.05 in round 2 were retained in the multi‐variable model. In the fourth round, all factors dropped in round 3 were added back in to the model one by one and all variables with *P* < 0.05 were retained, giving the final models. The study‐long and visit‐by‐visit models were then run with household ID and both household and individual ID as random effects respectively, to account for clustering and factors that were no longer significant (*P* ≥ 0.05) were dropped.

Acquisition was assessed over the full study period (study‐long) and visit‐by‐visit. Individuals were defined as positive for study‐long acquisition if they had a negative swab (no meningococci isolated) at visits 0 or 1 and a positive swab (any meningococci isolated) at any following visit. Individuals were defined as negative for study‐long acquisition if they had a negative swab at visits 0 or 1 and no positive swab at any subsequent visit. Individuals with three or more missed visits in total were excluded, as the possibility of acquisition during this missed period could not be ruled out and individuals carrying at visits 0 or 1 were also excluded.

Individuals were defined as positive for visit‐by‐visit acquisition on a given visit if the individual had a positive swab at the current visit and a negative swab at the previous visit or carried a different strain at the previous visit and the strain was not previously carried during the study. Strains were assessed by genogroup and porA variable regions 1 and 2. Individuals were defined as negative for visit‐by‐visit acquisition on a given visit if the individual had a negative swab at the previous visit and a negative swab at the current visit. Individuals carrying an identical strain to that obtained at the previous visit and individuals who cleared carriage were excluded from the analysis. Tables S1 and S2 provide the classification of cases for study‐long and visit‐by‐visit acquisition.

We defined the dry season as January to May and the rainy season as June to December. Because we found a significant association between sore throat and season and also previous studies have demonstrated an interaction between meningococcal carriage, upper respiratory tract infection and season, we also tested for interaction between sore throat and season in our final model and found that the model with an interaction term fitted better than the model with no interaction (Table S4).

### Ethics

The study was approved by the ethics committee of the London School of Hygiene and Tropical Medicine and by the relevant ethical authorities in each African centre 9. The head of the household or another responsible adult gave verbal informed consent for the household to be included in the study. Each individual recruited within that household gave written informed consent; for children under the age of 18 years a parent or guardian gave written consent and children aged over 12 years were additionally asked to give written assent.

## Results

### Acquisition over course of the study

Overall, 169/861 (20%) of the non‐index carriers became pharyngeal carriers of a meningococcus at least once over the course of the study. A higher proportion of 5‐ to 14‐year‐olds acquired carriage than other age groups and a higher proportion of participants acquired carriage in Senegal, Niger, Ghana and Ethiopia relative to Chad and Mali (Table 1). A wide variation in acquisition rates was observed between countries. Genogroup W and capsule‐null (cnl) meningococci accounted for the majority (83%) of acquisitions. The acquisition rates of genogroup W meningococci were 2.0% per month (95% CI 1.6–2.4) double that of cnl meningococci at 1.0% per month (95% CI 0.74–1.4). Genogroups A, C, Y and other genogroup (i.e. other than A, B, C, W, X, Y or cnl) acquisitions were uncommon and no genogroup B or X acquisitions were detected.

**Table 1:** Risk factors for *Neisseria meningitidis* acquisition over the full study period: single risk factor analysis and multi‐variable model. Adjustment was made in both single and multi‐variable analysis for age, country and sex

Factor	Single risk factor analysis				Multi‐variable model			
	Total	Positive (%)	OR	95% CI	Total	Positive (%)	OR	95% CI
Age								
30 plus					205	11.7	1	
Under 5					91	28.6	3.12	(1.27, 8.05)
5–14					108	23.1	3.62	(1.42, 9.93)
15–29					161	21.1	2.38	(1.22, 4.76)
Country								
Chad					54	5.6	1	
Ethiopia					64	26.6	7.65	(1.81, 44.4)
Ghana					74	23	6.77	(1.52, 40.1)
Mali					157	5.7	0.532	(0.110, 3.22)
Niger					206	28.6	10.0	(2.53, 57.3)
Senegal					10	40	13.3	(1.23, 159)
Sex								
Female					326	17.5	1	
Male					239	21.8	1.00	(0.585, 1.71)
Exposure to wood smoke in house (apart from use in cooking)^a^								
No	372	20.2	1		261	19.2	1	
Yes	478	19.0	2.74	(1.76, 4.32)	304	19.4	2.60	(1.26, 5.59)
Tobacco exposure^a^								
None	234	14.1	1		230	13.5	1	
Passive (secondhand) smoke	312	22.8	1.92	(0.965, 3.77)	312	22.8	1.92	(0.823, 4.55)
Active smoker	23	30.4	3.75	(1.23, 10.8)	23	30.4	3.57	(0.978, 13.0)^b^
Any sore throat reported^a^								
No	651	17.8	1					
Yes	208	25.5	1.66	(1.09, 2.53)				
Any runny nose reported^a^								
No	184	20.7	1					
Yes	675	19.4	1.57	(0.995, 2.51)				
Use gas as primary cooking fuel^a^								
No	832	20.0	1					
Yes	25	12.0	0.311	(0.0664, 1.03)				
Completion of primary school (amongst over 17 years)^a^								
No	269	18.2	1					
Yes	99	11.1	0.381	(0.170, 0.793)				
Household member completed secondary school^a^								
No	444	22.3	1					
Yes	415	16.9	0.670	(0.455, 0.983)				
More than 2 participants per room^a^								
No	484	14.5	1					
Yes	375	26.4	1.44	(0.996, 2.10)				
Attending primary school (ages 5–17)								
No	52	25	1					
Yes	254	23.2	0.721	(0.325, 1.65)				
Regular social meetings								
None	202	20.3	1					
1–2 per week	68	16.2	0.916	(0.404, 1.96)				
3–4 per week	48	8.3	0.531	(0.141, 1.61)				
5–7 per week	52	5.8	0.356	(0.0793, 1.14)				
Index carrier in household								
No	259	12.0	1					
Yes	600	23.0	1.32	(0.826, 2.16)				
Use wood as primary cooking fuel								
No	31	12.9	1					
Yes	828	19.9	1.02	(0.340, 3.83)				
Indoor kitchen								
No	660	16.4	1					
Yes	199	30.7	1.28	(0.838, 1.94)				

NB Total number of individuals may not sum to 861 in every case because of missing values.

a *P*‐value less than 0.1 in single risk factor analysis.

b *P*‐value less than 0.05.

In the final multi‐variable model, the highest odds of acquisition were amongst 5‐ to 14‐ year olds, with odds in all age groups under 30 years of age being significantly higher than the reference group of individuals 30 years and older (Table 1). Active smokers had higher odds of acquiring carriage than non‐smokers living in households with no smokers, with a lower confidence bound just below 1 (OR 3.57 95% CI 0.98–12.99). Non‐smokers living in households with smokers also had elevated odds of acquisition but the difference was not statistically significant. Wood was the ubiquitous cooking fuel, with 96% of the participants using this as cooking fuel; 56% of the participants had additional wood smoke exposure. Participants with household exposure to wood smoke (independent of using wood as cooking fuel) had higher odds of acquiring carriage than those without (OR 2.60 95% CI 1.26–5.59). Although this trend was not significant in the regression analysis, higher acquisition rates were observed in households with an indoor kitchen and in households which used wood as the primary cooking fuel than in those who did not.

### Visit‐specific acquisition analysis

Participants who said they had a sore throat since the previous visit during the dry season were significantly more likely (OR 3.67 95% CI 1.95–6.65) to have acquired carriage in that time period than those who did not have a sore throat in the rainy season (Table 2). Those who reported taking antibiotics since the previous visit were significantly less likely (OR 0.169 95% CI 0.0271–0.564) to have acquired carriage.

**Table 2:** Risk factors for visit‐by‐visit *Neisseria meningitidis* acquisition: single risk factor analysis and multi‐variable model. Adjustment was made *a priori* in both single and multi‐variable analysis for age, country and sex

Factor	Single risk factor analysis (plus *a priori*)				Multi‐variable model			
	Total	Positive (%)	OR	95% CI	Total	Positive (%)	OR	95% CI
Age								
30 plus					1504	1.8	1	
Under 5					1539	3.4	1.99	(1.22, 3.32)
5–14					2129	4.2	2.76	(1.75, 4.48)
15–29					1239	3	1.83	(1.08, 3.15)
Country								
Chad					990	0.6	1	
Ethiopia					564	4.6	7.54	(2.59, 24.5)
Ghana					828	3.5	5.7	(1.96, 18.6)
Mali					1574	0.9	1.51	(0.483, 5.13)
Niger					2281	5.2	11.5	(4.53, 34.5)
Senegal					174	7.5	14.2	(3.6, 60.7)
Sex								
Female					3405	2.9	1	
Male					3006	3.6	1.23	(0.907, 1.68)
Antibiotic taken^a^								
No	6592	3.5	1		6150	3.3	1	
Yes	261	0.8	0.197	(0.0323, 0.623)	261	0.8	0.169	(0.0271, 0.564)
Interaction term^a^								
No sore throat, rainy	2643	3.3	1		2643	3.3	1	
No sore throat, dry	3481	2.8	0.88	(0.651, 1.19)	3481	2.8	0.844	(0.617, 1.16)
Sore throat, rainy	123	2.4	0.906	(0.218, 2.52)	123	2.4	0.82	(0.192, 2.39)
Sore throat, dry	164	11	3.72	(2.09, 6.34)	164	11	3.67	(1.95, 6.65)
Sore throat^a^								
No	6566	3.3	1					
Yes	287	7.3	2.64	(1.58, 4.19)				
Season								
Rainy: June to December	1944	3.1	1					
Dry: January to May	4467	3.3	1.07	(0.78, 1.47)				
Meningitis vaccination								
No	5743	3.7	1					
Yes	1110	2	1.54	(0.899, 2.55)				
Attendance at social event								
No	3319	4.4	1					
Yes	3534	2.5	0.851	(0.63, 1.14)				
Travel greater than one hour								
No	6055	3.6	1					
Yes	798	2	0.955	(0.538, 1.58)				
Cough								
No	5163	3.6	1					
Yes	1690	3	0.955	(0.682, 1.31)				
Runny nose								
No	4634	3.8	1					
Yes	2219	2.6	0.961	(0.689, 1.32)				

a *P*‐value less than 0.1 in single risk factor analysis.

## Discussion

This longitudinal study found a higher risk of acquisition amongst individuals who reported a sore throat since the previous visit, but only during the dry season. An association between an upper respiratory tract infection and meningococcal carriage has been reported previously 14. A sore throat could be due to an initial inflammation of the pharynx from meningococcal colonisation or could be caused by a concurrent unrelated infection that predisposes an individual to acquisition 15. If the latter is true, upper respiratory tract infections in combination with dust and low humidity may be an important driver for the high risk of meningitis epidemics in the dry season. This hypothesis is supported by a recent study indicating an association between upper respiratory tract infection (defined as otitis, severe sore throat and rhinopharyngitis) and meningitis outbreaks in Burkina Faso 16. Such upper respiratory tract infections could plausibly increase both the risk of acquisition and the risk of invasion after acquisition.

The 5 to 14‐year‐old age group had the highest acquisition rate. The highest prevalence of carriage in cross‐sectional MenAfriCar studies and in Burkina Faso in 2009 was similarly highest in 5 to 14‐year olds 7, 17. An overall acquisition rate of 2.4% (95% CI 1.6–4.0%) per month was estimated from this same study using a hidden Markov model 9. There were no significant differences reported by age group, but data were subdivided by control and index households and there was no adjustment for other risk factors.

Additional factors linked to acquisition of meningococci over the course of this study were smoking tobacco and exposure to wood smoke. Smoking, passive exposure to smoke and to smokers has been shown to convey a high risk of carriage and invasive disease in high‐income countries 18, 19, 20, 21. Exposure to cigarette smoke has also been linked to the risk of carriage in the meningitis belt 7, 14. The higher risk of acquisition from smoke exposure in this study suggests a direct risk from smoke itself, potentially from interference with mucosal immunity, as exposure to wood smoke was an independent risk factor. Exposure to smoke from wood fires has also been shown as a risk factor for meningococcal meningitis in northern Ghana 22. Although use of wood as primary cooking fuel was not found to be a significant risk factor, this could be explained by the fact that nearly all study participants relied on wood as primary fuel or that some households used outdoor kitchens, thus moderating the degree of exposure.

Strengths of this study are the multi‐centre design across seven countries of the meningitis belt conducted at the same time, including a mix of urban and rural populations with a broad age range, the use of standardised field and laboratory protocols and a large sample size. Measuring acquisition rather than carriage ensures that the risk factors identified in this study are not biased by factors associated with longer carriage duration. A comprehensive range of risk factors was included, so that important confounding factors are unlikely to have been missed; however, the sampling of carriers and non‐carriers was not random and we would expect some misclassification of carriage status from the known low sensitivity of pharyngeal swabbing.

Both the acquisition of meningococci found in this longitudinal study and prevalence of carriage in the MenAfriCar cross‐sectional studies varied considerably by country. Although laboratory methods were standardised across centres, differences in laboratory techniques could still have contributed to some of the differences observed. As most meningococcal acquisitions were either genogroup W or capsule‐null and outside epidemics, it cannot be assumed that risk factors for acquisition of other genogroups or during epidemics would be the same as those found in this study 23.

It was surprising that some risk factors such as household crowding that have long been known to raise the risk of carriage and disease 7, 13, 24, 25 were not associated in this study with acquisition. Crowding was measured here by numbers sharing a bedroom or bedmat and by numbers of people per room in the household. It is possible that crowded living conditions are so prevalent across the meningitis belt countries that any effect of crowding on acquisition is not detectable. A study in rural Gambia did not find any differences in crowding between compounds with and without cases of meningococcal meningitis during an epidemic 26.

Reported vaccination was clustered in particular time periods and countries corresponding to the introduction of group A conjugate vaccine. Vaccination was not found to be protective against carriage acquisition. However, we would not expect a group A conjugate vaccine to have a significant impact on carriage in this study as very few group A carriers were detected.

We were not able to draw any conclusions regarding the relationship between carriage acquisition and disease incidence because none of the study sites reported an outbreak of meningitis during the follow‐up period.

This study involved multiple countries and examined an exhaustive set of household and individual risk factors for meningococcal acquisition. The importance of identifying these risk factors is that acquisition is a necessary prerequisite for invasive disease. Acquisition studies also play a potential role in vaccine evaluation. Of particular interest for countries of the African meningitis belt is the finding that symptoms of upper respiratory tract infection are linked to risk of acquisition, but only in the dry season. The evidence is mounting that such infections are an important factor behind the risk of epidemics in the meningitis belt.

## Supporting information


**Table S1.** Case definition for study‐long acquisition.
**Table S2**. Case definition for visit‐by‐visit acquisition.
**Table S3.** Odds of sore throat adjusting for age, country, sex and season.
**Table S4.** Likelihood ratio test comparing visit‐by‐visit model with and without term of interaction between season and sore throat.

## Appendix 1

Institutions and individual members of the MenAfriCar consortium who contributed to this study. Armauer Hansen Research Institute, Addis Ababa, Ethiopia: Oumer Ali, Abraham Aseffa (PI), Ahmed Bedru, Tsehaynesh Lema, Tesfaye Moti, Yenenesh Tekletsion, Alemayehu Worku, Haimanot Guebre Xabher (deceased), Lawrence Yamuah. Centre de Recherche Médicale et Sanitaire (CERMES), Niamey, Niger (Member of the International Network of Pasteur Institutes): Rahamatou Moustapha Boukary, Jean‐Marc Collard (PI), Ibrahim Dan Dano, Ibrahim Habiboulaye, Bassira Issaka, Jean‐François Jusot, Sani Ousmane, Issoufa Rabe. Centre de Support en Santé International (CSSI), N'Djamena, Chad: Doumagoum Moto Daugla (PI), Jean Pierre Gami, Kadidja Gamougam, Lodoum Mbainadji, Nathan Naibei, Maxime Narbé, Jacques Toralta. Centre pour les Vaccins en Développement, Bamako, Mali: Abdoulaye Berthe, Kanny Diallo, Mahamadou Keita, Uma Onwuchekwa, Samba O. Sow (PI), Boubou Tamboura, Awa Traore, Alou Toure. Centers for Disease Control, Atlanta, USA: Tom Clark, Leonard Mayer. Department of Community Medicine, University of Maiduguri, Maiduguri, Nigeria: Mary Amodu, Omeiza Beida, Galadima Gadzama, Babatunji Omotara (PI), Zailani Sambo, Shuaibu Yahya. Faculty of Infectious Disease, London School of Hygiene & Tropical Medicine, London, UK: Daniel Chandramohan, Brian M. Greenwood (PI), Musa Hassan‐King, Olivier Manigart, Maria Nascimento, James M. Stuart, Arouna Woukeu. Princeton University, USA: Nicole E. Basta. Public Health England Vaccine Evaluation Unit, Manchester, UK: Xilian Bai, Ray Borrow, Helen Findlow. Institut de Recherche pour le Développement, Dakar, Senegal: Serge Alavo, Hubert Bassene, Aldiouma Diallo (PI), Marietou Dieng, Souleymane Doucouré, Jules François Gomis, Assane Ndiaye, Cheikh Sokhna, Jean François Trape. Navrongo Health Research Centre, Navrongo, Ghana: Bugri Akalifa (deceased), Abudulai Forgor (deceased), Abraham Hodgson (PI), Isaac Osei, Stephen L. Quaye, John Williams, Peter Wontuo. University of Bristol, UK: Thomas Irving. University of Cambridge, UK; Caroline L. Trotter. University of Oxford, UK: Julia Bennett, Dorothea Hill, Odile Harrison, Martin C.J. Maiden, Lisa Rebbetts, Eleanor Watkins.
