# Supplementary material for: Risk factors for acquisition of meningococcal carriage in the African meningitis belt
Source: Trop Med Int Health. 2019 Feb 6;24(4):392–400. doi: 10.1111/tmi.13203 (PMC6563094; doi:10.1111/tmi.13203)
Supplement: Supplementary file 1 — Table S1. Case definition for study‐long acquisition. Table S2. Case definition for visit‐by‐visit acquisition. Table S3. Odds of sore throat adjusting for age, country, sex and season. Table S4. Likelihood ratio test comparing visit‐by‐visit model with and without term of interaction between season and sore throat. [file TMI-24-392-s001.docx]

**Table S1.** Case definition for study-long acquisition.

| Classification | Carriage at visits 0 or 1 | Carriage at visits 2-9 | More than 3 missed visits | Number of individuals |
| --- | --- | --- | --- | --- |
| Not acquisition | No | No | No | 692 |
| Acquisition | No | Yes | No | 169 |
| Excluded | No | No | Yes | 231 |
| Excluded | No | Yes | Yes | 18 |
| Excluded | Yes | No | No | 42 |
| Excluded | Yes | No | Yes | 18 |
| Excluded | Yes | Yes | No | 159 |
| Excluded | Yes | Yes | Yes | 22 |

**Table S2**. Case definition for visit-by-visit acquisition.

| Classification | Carriage at previous visit | Carriage at current visit | Strain previously observed | Number of visit pairs |
| --- | --- | --- | --- | --- |
| Not acquisition | No | No | Not applicable | 6768 |
| Acquisition | No | Yes | No | 226 |
| Acquisition | Yes | Yes | No | 47 |
| Excluded | No | Yes | Yes | 183 |
| Excluded | Yes | Yes | Yes | 366 |
| Excluded | No | No data | Not applicable | 1180 |
| Excluded | Yes | No | Not applicable | 516 |
| Excluded | Yes | No data | Not applicable | 128 |
| Excluded | No data | No | Not applicable | 1677 |
| Excluded | No data | Yes | Not applicable | 304 |
| Excluded | No data | No data | Not applicable | 2115 |

**Table S3.** Odds of sore throat adjusting for age, country, sex and season.

| Factor | Total | Percent reporting sore throat | OR | 95% CI |
| --- | --- | --- | --- | --- |
| Age |  |  |  |  |
| 30 plus | 2024 | 6.6 | 1 |  |
| Under 5 | 1914 | 2.4 | 0.372 | (0.261,0.522) |
| 5-14 | 2806 | 2.6 | 0.399 | (0.295,0.534) |
| 15-29 | 1752 | 7.4 | 1.13 | (0.873,1.45) |
| Country |  |  |  |  |
| Chad | 1038 | 5.8 | 1 |  |
| Ethiopia | 940 | 7.1 | 1.27 | (0.883,1.83) |
| Ghana | 1135 | 8.6 | 1.41 | (1.01,1.98) |
| Mali | 1854 | 1.1 | 0.174 | (0.101,0.287) |
| Niger | 3179 | 4.3 | 0.728 | (0.532,1.01) |
| Senegal | 350 | 0 |  |  |
| Sex |  |  |  |  |
| Female | 4424 | 4.3 | 1 |  |
| Male | 4072 | 4.6 | 1.14 | (0.92,1.4) |
| Season |  |  |  |  |
| Rainy: June to December | 3617 | 4.4 | 1 |  |
| Dry: January to May | 4879 | 4.5 | 1.27 | (1.02,1.57) |

**Table S4.** Likelihood ratio test comparing visit-by-visit model with and without term of interaction between season and sore throat.

|  | | | | | | Chi-square | | |
| --- | --- | --- | --- | --- | --- | --- | --- | --- |
| Model | Degrees of freedom | AIC | BIC | Log-likelihood | Deviance | Statistic | Degrees of freedom | p-value |
| Acquisition ~ Age + Country + Sex + Sore throat + Antibiotic | 14 | 1660 | 1754 | -815.82 | 1631.6 |  |  |  |
| Acquisition ~ Age + Country + Sex + Sore throat + Antibiotic + Season | 15 | 1662 | 1763 | -815.77 | 1631.5 | 0.099 | 1 | 0.75 |
| Acquisition ~ Age + Country + Sex + Sore throat + Antibiotic + Season + Season and sore throat interaction | 16 | 1656 | 1764 | -811.97 | 1623.9 | 7.6 | 1 | 0.006 |
